# CPR initiated after telephone-assisted instruction produces a better outcome of bystander-witnessed out-of-hospital cardiac arrests than no bystander CPR but is less effective than CPR on the bystander's own initiative

**DOI:** 10.1186/cc10877

**Published:** 2012-03-20

**Authors:** H Inaba, T Kamikura, K Takase, W Omi, S Sakagami, Y Myojo, J Taniguchi

**Affiliations:** 1 Kanazawa University Graduate School of Medicine, Kanazawa, Japan; 2 Kanazawa Medical Center, Kanazawa, Japan; 3 Ishikawa Prefectural Central Hospital, Kanazawa, Japan

## Introduction

Telephone CPR has been shown to increase the incidence of bystander CPR and is expected to improve the outcomes of out-of-hospital cardiac arrests (OHCAs). The aim of present study was to clarify if the outcomes of bystander-witnessed OHCAs having CC-only and conventional CPR following telephone CPR may be better than those having no bystander CPR and if the type (CC-only and conventional) and origin (following telephone CPR and on bystander's own initiative) may affect the outcomes of bystander-witnessed OHCAs with bystander CPR.

## Methods

From the Japanese nationwide database for 431,968 OHCAs that occurred from January 2005 to December 2008, we extracted and analyzed 112,144 bystander-witnessed OHCAs without any involvement of physicians, using multiple logistic regression analysis.

## Results

The analysis for all bystander-witnessed OHCAs revealed that both CC-only and conventional CPR following telephone CPR produce better outcomes than no bystander CPR (Table 1). The analysis for bystander-witnessed OHCAs with bystander CPR disclosed that CPR on the bystander's own initiative produces a better outcome than CPR following telephone CPR (Table 2).

**Table 1:** Comparison of survival between OHCAs without bystander CPR and bystander CPR in bystander-witnessed OHCAs

Factor	Adjusted odds ratio	95% CI
Type of CPR		
No bystander CPR	Reference	
CC-only CPR following telephone-CPR	1.66	1.49 to 1.84
Conventional CPR following telephone-CPR	1.67	1.48 to 1.89
CC-only CPR on bystander's own initiative	2.22	1.99 to 2.49
Conventional CPR on bystander's own initiative	2.36	2.10 to 2.66
Aetiology		
Presumed cardiac	2.44	2.27 to 2.63
Noncardiac	Reference	
Time intervals		
Witness-call	0.98	0.97 to 0.98
Witness-first CPR performed either by	0.97	0.96 to 0.98
citizens or by EMTs		
Call-arrival at patients	0.93	0.92 to 0.94

Comparisons of 1-month survival with favourable neurological outcomes between OHCAs without bystander CPR and with four types of bystander CPR in bystander-witnessed OHCAs (multiple logistic regression analysis).

**Table 2:** Effects of type and origin by bystander CPR on survival of bystander-witnessed OHCAs having bystander CPR

Factor	Adjusted odds ratio	95% CI
Type of CPR		
CC-only CPR	0.96	0.88 to 1.04
Conventional CPR	Reference	
Origin of CPR		
Following telephone-CPR	0.73	0.67 to 0.80
On bystander's own initiative	Reference	
Aetiology		
Presumed cardiac	2.27	2.05 to 2.51
Noncardiac	Reference	
Time intervals		
Witness-call	0.99	0.98 to 0.99
Witness-first CPR performed either by	0.98	0.97 to 0.99
citizens or by EMTs		
Call-arrival at patients	0.88	0.87 to 0.90

Effects of type and origin by bystander CPR on 1-month survival with favourable neurological outcomes of bystander-witnessed OHCAs having bystander CPR (multiple logistic regression analysis).

## Conclusion

Telephone CPR improves the outcomes of bystander-witnessed OHCAS. However, efforts to increase the incidence of early CPR on the bystander's own initiative would be necessary to obtain a higher incidence of survival in bystander-witnessed OHCAs.

